# Dr. Wile’s Death

**Published:** 1887-05

**Authors:** W. S. Elkin, James A. Gray, N. O. Harris


### DR. WILE'S DEATH.

Dr. Henry Wile, who left this city two weeks ago for Denver, Col., found the climate of that State more injurious than beneficial to his health. After arriving there he was much prostrated, and realizing that death was near at hand, he left at once for Rochester, N. Y., the home of his parents. He had hoped to reach thatcity before the end came, but he died on Monday, April 11th, on the train between Chicago and Rochester. Dr. Wile was twenty-eight years of age, a graduate of the University of Rochester in 1879, and of the medical department of the University of Pennsylvania in 1882. He subsequently visited Europe and pursued his studies in Berlin, Vienna and Paris, making skin diseases a specialty. He was a hard student and a gifted writer, and at the time of his death had in course of preparation a book on skin diseases. He had gained the esteem and confidence of the medical profession of this city, and was building up a reputation as an expert.

At a meeting of the Atlanta Society of Medicine, held April 26th, 1887, the following expressions regarding the death of Dr. Henry Wile were unanimously adopted:

In the death of Dr. Henry Wile our Society has sustained an irreparable loss of one of its most worthy and brightest members. For two years he has been a member of our body, and until a few weeks ago was ever active in the performance of all the duties that fell upon him. The papers he presented to the Society were literary gems, containing an almost unbounded amount of scientific knowledge and valuable information. His great medical attainments, wise counsel and sober judgment will not only be missed by the members of our Society, but by the profession at large, who, from reading his gifted articles published in the leading journals of this country, have long since become conscious of his skill and reputation as an expert in the practice of skin diseases. Besides his valuable store of medical and scientific knowledge, he possessed a kind heart and an evenness of temper that drew all near and made many warm and lasting friends.

We further desire that these expressions of heartfelt sympathy be conveyed to his bereaved family and spread upon the minutes of the Society.

W. S. ELKIN,
JAMES A. GRAY,
N. O. HARRIS.

### ITEMS.

---

DR. GEORGE H. NOBLE, for a number of years a partner of Dr. V. H. Taliaferro, of this city, has removed to Anniston, Ala.

DR. F. W. MCRAE, of this city, has gone to New York, where he will remain for several months attending the New York Polyclinic.

WE have received from Parke, Davis & Co., of Detroit, a portrait of the eminent Dr. Robert Koch. This picture will be sent free to any physician on application to Parke, Davis & Co.

THE fifteenth annual meeting of the American Public Health Association will be held in Memphis, Tennessee, November 8th, 9th, 10th and 11th, 1887. Dr. W. H. Elliott, of Savannah, is a member of the advisory council.

THE thirty-eighth annual session of the American Medical Association will be held in Chicago on Tuesday, Wednesday, Thursday and Friday, June 7, 8, 9 and 10, commencing on Tuesday at 11 a.m.

DR. J. MCF. GASTON, of this city, requests us to say that members of the medical profession who have treated cases of perforation of the vermiform appendix, or made *post mortem* examinations of such results, would confer a favor by reporting same to him without delay.

DR. HARRY HUZZA, formerly of this city, has recently associated himself with Dr. Robert Battey, of Rome, and will hereafter make that place his home. Dr. Huzza graduated at the last session of Jefferson Medical College with distinction. He is one of the brightest young men in Georgia.

In *Scribner's* for May, J. Macdonald Oxley describes Sable Island in an illustrated article bearing the title of "An Ocean

Graveyard," the fitness of which is more than proved by the curious "wreck-chart" accompanying the article, on which is recorded the long list of vessels that have met shipwreck and disaster on its barren sands.

NOTICE TO DELEGATES TO THE AMERICAN MEDICAL ASSOCIATION.—The East Tennessee, Virginia and Georgia Railway will give reduced rates to delegates to the American Medical Association, which meets in Chicago, June 7th. A number of the delegates will leave Atlanta in a special sleeper, at 1 p. m., June 5th. Delegates desiring rates and sleeping car accommodations should write at once to L. J. Ellis, Assistant General Passenger Agent, Atlanta, Ga.

The Western and Atlantic Railroad will also give delegates reduced rates. A parlor car will leave Atlanta by this line at 7:50 o'clock on the morning of June 6th, arriving in Chicago at 10.30 a. m. on the 7th. Delegates can address J. H. Griffin, 28 Wall street, Atlanta, Ga., for rates and sleeping car accommodations.

FLORIDA RAILWAY AND NAVIGATION CO.—Among the new advertisements in this issue of THE JOURNAL will be found one of this company. They own and control nearly six hundred miles of railroad in Florida, going into every portion of the State. Their passenger service is unsurpassed. Parties visiting Florida should see that their tickets read via F. R. & N.

HYDRONAPHTHOL, the latest antiseptic and disinfectant obtained from the phenol series of coal tar products, is rapidly forging its way to the front in popular estimation. Surgeons and gynæcologists everywhere, in both hospital and private practice, accord it the highest possible merit for all the purposes of an antiseptic and disinfectant. Its freedom from odor, poisonous properties or corrosiveness gievs it preference over carbolic acid and all other antiseptics heretofore employed. A book, fully describing hydronaphthol, will be mailed free to physicians sending their address to Seabury & Johnson, manufacturing chemists, 21 Platt street, New York.

COCA AS A CARDIAC TONIC.—The N. Y. *Medical Record* of February 26th, 1887, gives an interesting article entitled "Heart Strain and Weak Heart," by Beverly Robinson, M. D. We extract the following: "On several occasions, when digitalis has proved to be useless or injurious, I have had very excellent results from caffeine or convallaria. Certainly the latter drug is more easily tolerated by a sensitive stomach than digitalis is; and whenever the nervous supply of the heart is especially implicated, I believe that I secure more quieting effects from its employment. Among well-known cardiac tonics and stimulants for obtaining temporary good effects, at least, I know of no drug quite equal to coca. Given in the form of wine or fluid extract, it does much at times to restore the heart-muscle to its former tone. I have obtained the best effects from the use of Mariani's wine. From personal information given me by this reliable pharmacist, these results are attributable to the excellent quality of the coca leaves and of the wine which he uses in its manufacture."

MELLIN'S FOOD.—Prof. Dr. R. Fresenius, of Wiesbaden, Germany, has made an analysis of Mellin's food for infants and invalids, of which the following is a summary:

| | |
|---|---|
| Total carbohydrates | 72.56 |
| albuminoids | 9.75 |
| salts | 4.37 |
| moisture | 13.32 |
| | 100.00 |

Starch and cane sugar, none; reaction, alkaline.

A copy of the detailed analysis and remarks of this first chemist in the world may be had by application to Messrs. Doliber, Goodale & Co., 41 and 42 Central wharf, Boston, Mass.

